# Antidepressant-like effects of extinction learning as an animal model of behavioral therapy

**DOI:** 10.1038/s41380-025-03380-8

**Published:** 2025-12-12

**Authors:** Jing Liu, Sarah E. Bulin, David A. Morilak

**Affiliations:** 1 Department of Pharmacology and Center for Biomedical Neuroscience, University of Texas Health Science Center at San Antonio, San Antonio, TX 78229 USA; 2 South Texas Veterans Health Care System, San Antonio, TX 78229 USA

**Keywords:** Neuroscience, Depression

## Abstract

Exposure-based behavioral therapy, the most effective treatment for posttraumatic stress disorder (PTSD), also reduces depressive symptoms. However, neurobiological mechanisms underlying the beneficial effects of exposure-based behavioral therapy on depression remain unknown. Our lab has established fear extinction as a rat model of exposure therapy to investigate the mechanisms underlying its therapeutic behavioral effects in chronically stressed rats. In this study, we demonstrated that extinction learning reduced immobility in the forced-swim test and reversed chronic stress-induced reduction in sucrose preference. Chemogenetic inactivation of pyramidal neurons in the ventral medial prefrontal cortex (vmPFC) prevented these antidepressant-like effects of extinction. Extinction learning enhanced synaptic plasticity, reflected by enhanced optogenetically-induced long-term potentiation of mPFC responses evoked by stimulation of the afferent input from the mediodorsal thalamus (MDT). These results suggest that activity-dependent neuroplasticity induced in vmPFC by extinction learning may contribute to its antidepressant-like effects after chronic stress.

## Introduction

Major depressive disorder (MDD) and posttraumatic stress disorder (PTSD) are complex psychiatric illnesses, which affect about 20 and 8% of the US population in their lifetime, respectively, and produce serious economic burden on society [1, 2]. PTSD and MDD are highly comorbid, with approximately 50% of patients with PTSD also having symptoms of depression [3]. Co-occurrence of PTSD with MDD is associated with greater symptom severity, higher levels of suicidality, and poor response to treatment compared to those diagnosed with either disorder alone [4–6]. However, the pathophysiology of these comorbid disorders is still poorly understood, and treatment for PTSD with MDD remains inadequate.

Exposure-based behavioral therapy is currently the most effective treatment for PTSD [7]. Exposure therapy is based on fear extinction, through which repeated exposure to a fear-provoking stimulus in a safe environment reduces maladaptive stress responses elicited by reminders of the stimulus [8]. Clinical studies show that exposure therapy also improves symptoms of co-occurring depression in PTSD patients [9, 10]. Behavioral therapy with exposure processing reduces depressive symptoms in patients with MDD by targeting experiential avoidance [11]. Therefore, understanding the neurobiological mechanisms by which exposure therapy exerts effects on depressive symptoms may inform more effective treatments for comorbid PTSD and MDD.

Chronic stress is a risk factor for many psychiatric conditions, including MDD and PTSD [12]. Chronic unpredictable stress (CUS) is used in rodents to investigate the pathophysiology of stress-related psychiatric disorders, and to inform new treatment strategies [13]. Our lab has previously established fear extinction as a rodent model for exposure therapy after CUS [14–16]. Fear extinction restored adaptive active coping in the shock probe test and improved cognitive flexibility after CUS, which model symptom dimensions of comorbid MDD and PTSD [14, 15]. The beneficial effects of extinction learning were prevented by inhibiting pyramidal cell activity and protein synthesis in the ventromedial prefrontal cortex (vmPFC) [14, 15], demonstrating that the vmPFC is an important substrate for the therapeutic effects of fear extinction as a model of exposure therapy.

Dysfunction of the PFC is also implicated in MDD. Studies indicate decreased volume of the PFC, along with decreased neuronal size and altered dendritic structure in patients with MDD [17, 18]. Reduced PFC activity and impaired cortical long-term potentiation (LTP)-like plasticity have been observed in patients with MDD [19, 20]. Antidepressant treatment increased cortical excitability associated with symptom improvement [21]. In rodents, CUS decreased spine density and dendritic elaboration of mPFC pyramidal neurons [22–25], and attenuated local field potentials (LFPs) evoked in mPFC by stimulation of the mediodorsal thalamus (MDT) [26, 27]. Optogenetically-induced long-term depression (opto-LTD) of this same pathway reproduced the MDD-like cognitive deficits induced by CUS [26]. By contrast, stimulation of the mPFC or optogenetic LTP of the MDT afferent to mPFC produced antidepressant-like effects [26]. We further showed that therapeutic effects of extinction on cognitive flexibility were reversed by blocking brain-derived neurotrophic factor (BDNF) signaling in the vmPFC of chronically stressed rats [16]. As BDNF is involved in synaptic plasticity [28], we hypothesize that extinction exerts its therapeutic effects in reversing stress-induced behavioral deficits by promoting or restoring functional and structural plasticity in the vmPFC.

In the current study, we first extended the characterization of extinction as a model of exposure therapy to include effects on other behavioral changes that model aspects of depression and depression comorbid with PTSD, including immobility on the forced swim test (FST), widely used as an indicator of antidepressant efficacy, and CUS-induced reduction in sucrose preference as a measure of anhedonia. Then we employed a chemogenetic approach to selectively inhibit pyramidal neurons in the vmPFC, (infralimbic (IL) and ventral prelimbic (PL) cortex), the functional homolog of human vmPFC implicated in depression [29, 30], to determine its role in the antidepressant-like effects of extinction learning. Further, we explored effects of extinction learning on CUS-induced changes in synaptic plasticity in the vmPFC measured by optogenetically-induced LTP in the afferent pathway from MDT to vmPFC. Portions of this work have been presented in abstract form [31, 32].

## Materials and methods

### Animals

Initial group size targets were estimated by power analysis: an estimated mean difference of 36% with standard deviation 24% (effect size = 1.5) will be detected at p < 0.05 by a two-tailed test with power = 0.90 with n = 12/group. 338 adult male (164) and female (174) Sprague–Dawley rats (Envigo, Indianapolis, IN) were housed in same-sex groups of 2-3 on a 12/12 h light/dark cycle (lights on at 0700 h) with food and water *ad libitum*. Rats were 225-249 g at the time of arrival and acclimated at least 1 week before experiments began. Behavioral tests were performed between 10:00-14:00 in procedure rooms adjacent to the housing room. All procedures were conducted in accordance with National Institutes of Health guidelines and approved by the Institutional Animal Care and Use Committee of the University of Texas Health Science Center at San Antonio. Wherever possible, experimenters were blind to treatment conditions of the animals being tested.

### Reagents

AAV5-CaMKIIa-hM4D(Gi)-mCherry (titer ≥ 4×10¹² vg/mL), AAV5-CaMKIIα-EGFP ( ≥ 4×10¹² vg/mL) and AAV5-CaMKIIα-ChETA-YFP (3 × 10^12^ vg/mL) viruses were purchased from Addgene (Watertown, MA), and stored in 10 µL aliquots at –80 °C. Clozapine N-oxide (CNO, Tocris Bioscience, Minneapolis, MN) was dissolved in dimethyl sulfoxide (DMSO) as a stock solution (200 mg/mL) and diluted in saline to 1 mg/mL immediately before use. CNO or vehicle was injected intraperitoneally (1 mL/kg), as previously described [15].

### Viral administration for chemo- and optogenetic manipulation of vmPFC

To prepare the rats for chemogenetic inhibition of glutamatergic pyramidal neurons in vmPFC, they were anaesthetized with isoflurane (4% induction, 1-2% maintenance) and placed in a stereotaxic frame (David Kopf Instruments, Tujunga, CA). AAV5-CaMKIIa-hM4D(Gi)-mCherry or the control AAV5-CaMKIIα-EGFP vector was injected bilaterally (0.5 µL/side at 0.05 µL/min) into vmPFC (from bregma: AP + 2.8, ML ± 0.5, DV − 4.5 mm; [33]) using a 33-gauge beveled needle with a 10 µL Nanofil syringe controlled by an ultra-microinjection pump (WPI Inc, Sarasota, FL). After injection, the needle was left in place for 5 min before withdrawing. Behavior was tested 3 weeks after virus injection. Viral expression was verified by mCherry or GFP fluorescence. To prepare the rats for optogenetic stimulation of MDT axon terminals in vmPFC, AAV5-CaMKIIα-ChETA-YFP virus was injected bilaterally (0.5 µL per side) into the MDT (from bregma: AP –2.5, ML ± 0.9, DV –4.6 mm) [33], as previously described [26]. Animals were tested at least 6 weeks after viral injection.

### Chronic unpredictable stress

CUS procedure was as described previously [14–16, 26]. Different acute stressors were applied at varying times each day for 14 days (males) or 21 days (females), to achieve similar behavioral effects [16, 26]. Stressors included 30 min restraint, 10 min tail pinch, 15 min warm swim, 10 min cold swim, 1 h shaking/crowding, 45 min social defeat, 24 h constant light, 24 h wet bedding, or 15 min footshock. After stress procedures, rats recovered for 1-2 h in a separate room before returning to housing. Control and stressed animals were singly housed throughout the stress protocol.

### Behavioral procedures

#### Fear Extinction

The day before fear conditioning, rats were habituated to two contexts in sound-attenuating cabinets for 15 min each. Context A was the conditioning chamber (30.5 × 25.4 × 30.5 cm; model H10–11R-TC, Coulbourn Instruments, Holliston, MA) with square metal walls and a metal grid floor attached to a shock generator (model H13–15). Context B was a different chamber with smooth green vinyl floor and circular vinyl walls. On the day of fear conditioning, rats received 4 pairings of tone (10 kHz, 75 dB, 20 s) coterminus with footshock (0.8 mA, 0.5 s) in context A (average intertrial interval = 120 sec). Tone control rats were treated identically, except no shock was delivered. Conditioned fear was defined as percent freezing during each tone, measured videographically (FreezeView software, ActiMetrics #ACT-100, Coulbourn Instruments). At the times specified in each experiment below, extinction learning was administered as a therapeutic intervention in Context B, consisting of 16 presentations of tone alone with no shock (average intertrial interval = 120 s) [14].

#### Forced swim test

FST consisted of a 15-min pretest swim and a 5-min test swim [34]. Rats were singly housed for 5 days prior to the pretest, for which rats were placed in a cylindrical tank (46 × 21 cm) filled to a depth of 30 cm with 23  °C water. The 5-min test was performed 48 h or 8 days after the pretest. Immobility was defined as floating with no purposeful active movements other than those necessary to keep the nose above the water. Behavior was recorded, and immobility time was analyzed using Solomon Coder beta 19.08.02 (András Péter, http://solomoncoder.com).

#### Sucrose preference test

The procedure was modified from a previous paper [35]. Rats were habituated to two leak-resistant water bottles (All Living Things®, PetSmart. Phoenix, AZ) for 5 days and to 1% sucrose for 2 days prior to testing. On testing day, two identical bottles filled with tap water or 1% sucrose were provided to each animal after 4 h water deprivation. Left-right position of the bottles were counterbalanced. Rats were allowed to drink freely for 1 h. Sucrose and water consumption were measured by weighing the bottles. Sucrose preference was defined as percent of total fluid consumed (100 x sucrose/total intake) during the 1 h test.

#### Locomotor Activity

Locomotor activity was measured using the open field locomotor system (Med Associates, Fairfax, VT) for 30 min. Distance traveled was analyzed in 2-min intervals using Activity Monitor software (Med Associates).

### Experiments

**Fig. 1:**
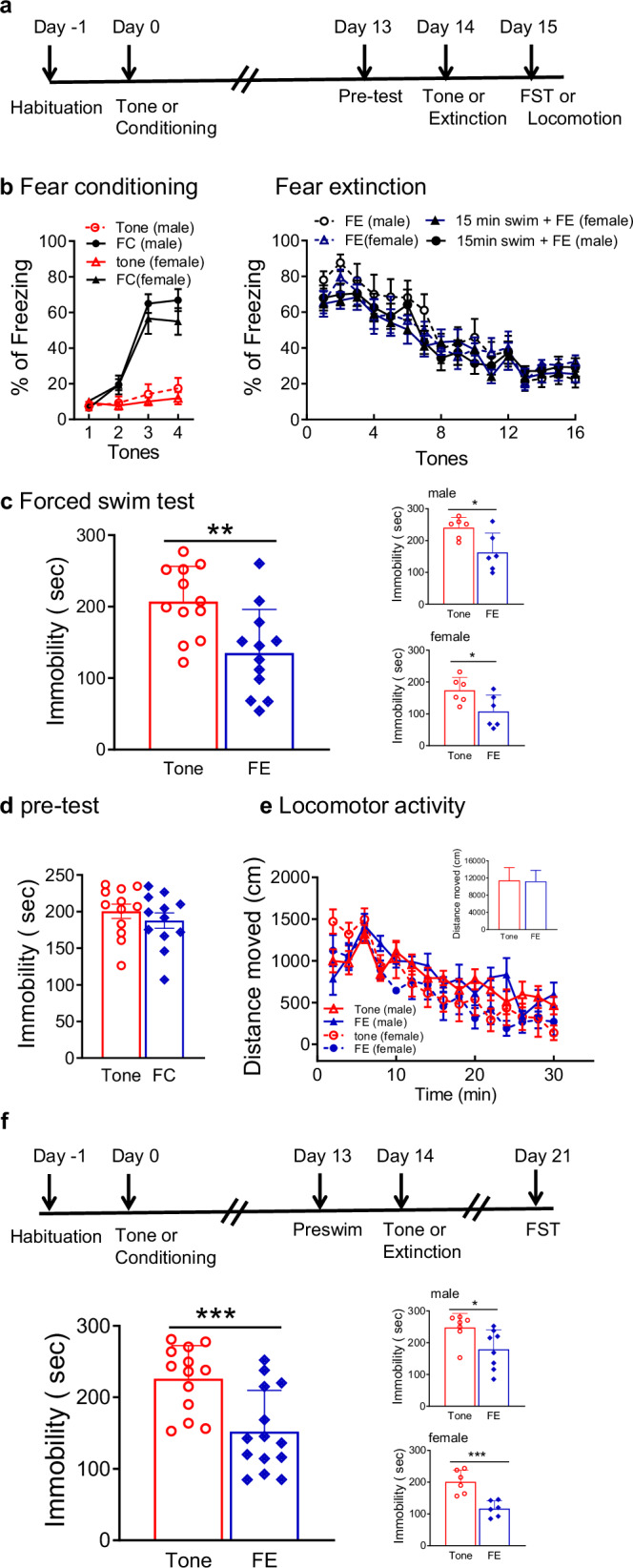
Antidepressant effects of extinction learning on forced swim test. (**a**) Timeline for experiments testing the effects of extinction on the FST. (**b**) Left panel: During fear conditioning (FC), freezing increased to approximately 60% after 4 tone-shock pairings. There was no difference between males and females (n = 16-17 males and 15 females per group). Right panel: Extinction curves were comparable for rats with or without exposure to the 15 min FST pre-swim 24 h prior to fear extinction (FE). There was no difference between males and females (n = 9-14 males and 9-12 females per group). (**c**) Extinction reduced immobility during the 5-min test swim of the FST. Insets show males and females separately (n = 12 per group, 6 males and 6 females) (**d**) There was no difference in immobility time in the first 5 min of the pre-swim for rats exposed to FC or tone control (n = 12 per group, 6 males and 6 females). (**e**) There were no effects of extinction on locomotor activity in the open field test, monitored for 30 min and analyzed in 2-min bins. Inset shows total distance traveled in the entire 30 min test (n = 13-14 per group, 7-8 males and 6 females). (**f**) Top: Timeline to test lasting effects of extinction on the FST. Bottom: Immobility on the FST was reduced 7 days after extinction. Insets show males and females separately (n = 13-14 per group, 7-8 males and 6 females). In all panels, data are expressed as mean ± SEM. *P < 0.05, **P < 0.01, ***P < 0.001.

**Effects of extinction on immobility in the forced swim test**. 96 rats (49 male and 47 female) were assigned to two groups (extinction vs tone control). Fourteen days after fear conditioning or tone control, extinction was administered in context B by presenting 16 tones without shock. Tone controls were treated identically, but because they had not experienced initial conditioning, no extinction learning occurred. The pre-swim was performed 24 h before extinction. In some rats, the test swim occurred 24 h after extinction (Fig. 1a). In other rats, to determine if antidepressant-like effects of extinction learning were long-lasting, test swim was 7 days after extinction (Fig. 1f).

**Fig. 2:**
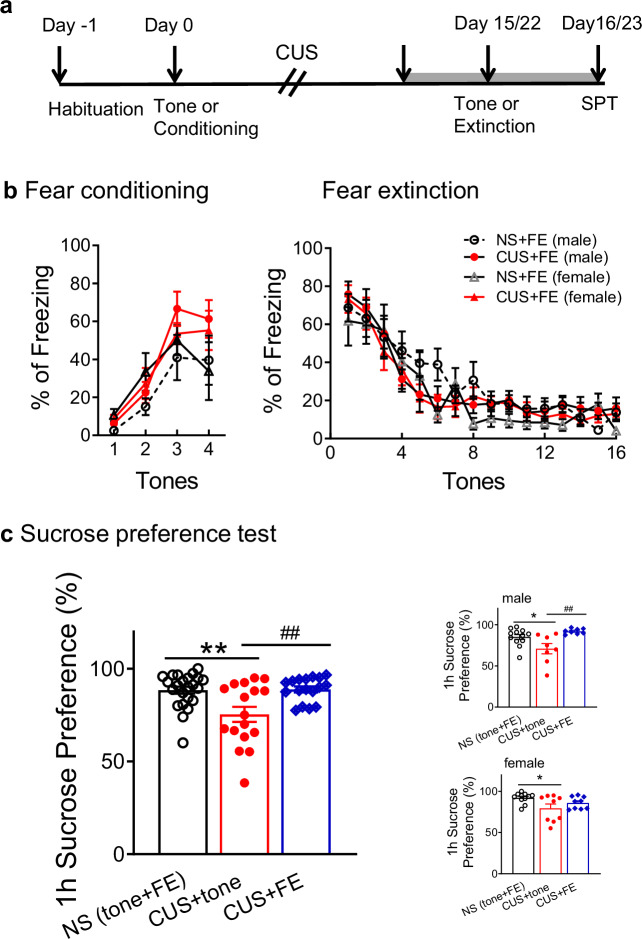
Effects of extinction learning on CUS-induced anhedonia. (**a**) Timeline for experiments testing the effects of extinction on CUS-induced reductions in sucrose preference. Gray bar indicates the habituation period for 1% sucrose. SPT: sucrose preference test. (**b**) Fear conditioning was comparable between groups prior to stress treatment. Likewise, fear extinction (FE) was comparable in both nonstressed and CUS-exposed rats, and in males and females (n = 12-17 per group, 6-8 males and 6-9 females). (**c**) CUS reduced sucrose preference, defined as percent of total fluid consumed (100 x sucrose /total intake) during the 1 h test period. Extinction reversed the effect of stress. Insets show male and female rats separately (n = 17-23 per group, 8-12 males and 9-11 females). Data are expressed as mean ± SEM. *P < 0.05, **P < 0.01 compared with NS control group; ^##^P < 0.01 compared with CUS + tone control group.

**Effects of extinction learning on sucrose preference**. 57 rats (28 male and 29 female) were assigned to 4 groups (CUS vs nonstressed (NS) x extinction vs tone control). CUS began the day after fear conditioning or tone control. Extinction was conducted 24 h after the last stressor; sucrose preference was tested 24 h after extinction (Fig. 2a).

**Fig. 3:**
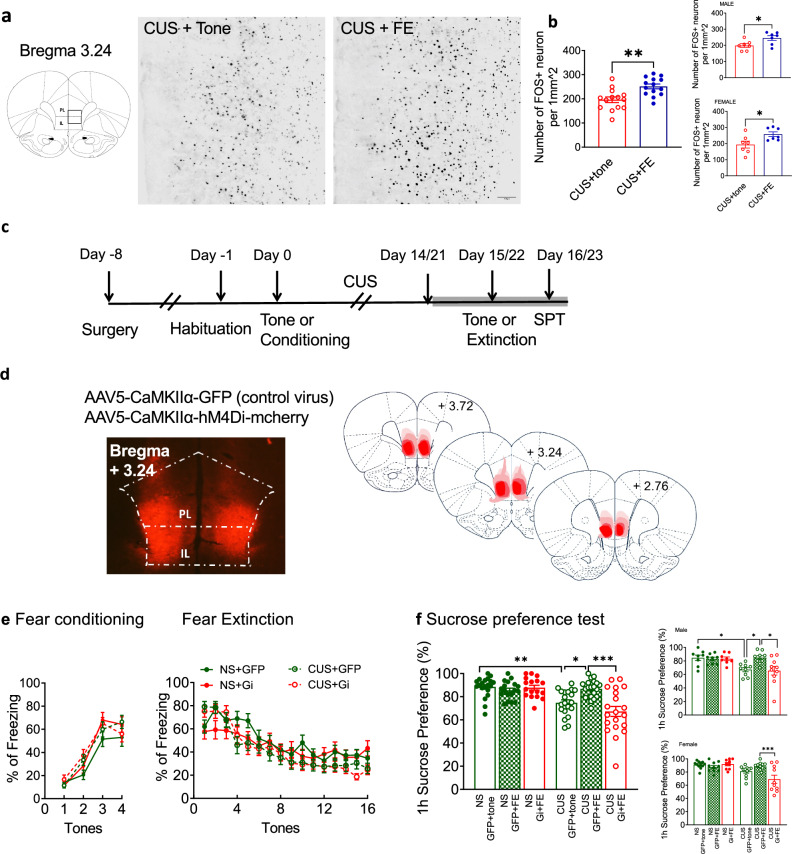
vmPFC activity is necessary for the antidepressant effects of extinction learning. (**a**) Extinction induced Fos expression in the vmPFC of stressed rats. Left: schematic illustration of the 1 mm^2^ region in which Fos-positive cells were counted in vmPFC. PL, prelimbic cortex; IL, infralimbic cortex. Right: Photomicrographs showing Fos immunolabel in the vmPFC of stressed rats after tone control exposure or extinction. (**b**) The number of Fos-positive neurons per mm^2^ was increased in the vmPFC of stressed rats after extinction compared to tone-control exposure. n = 14 per group, 7 males and 7 females. Insets show male and female rats separately. *P < 0.05, **P < 0.01. (**c**) Timeline for the DREADD experiments for which results are shown in panels d-f. SPT: sucrose preference test. (**d**) Injection sites for bilateral administration of the AAV-DREADD viruses. Left: Representative image of mCherry expression in vmPFC; Right: schematic illustration of injection sites. (**e**) Left: Fear conditioning in the rats with intra-vmPFC viral injections was comparable between groups prior to stress treatment. Right: Fear extinction performed 30 min after clozapine-N-oxide (CNO) injection was comparable to preceding experiments and did not differ between groups. (**f**) Sucrose preference measured 24 h after extinction. Neither extinction nor Gi DREADD activation with CNO had any effect on sucrose preference in nonstressed rats. CUS decreased sucrose preference (CUS + GFP+tone), and this was reversed by extinction (CUS + GFP + FE). The beneficial effect of extinction was prevented by Gi DREADD-mediated inhibition of pyramidal cells in the vmPFC during extinction (CUS+Gi+FE). n = 17-20 per group, 8-10 males and 9-12 females. Insets show male and female rats separately. Data expressed as mean ± SEM. *P < 0.05, **P < 0.01, ***P < 0.001.

**Effects of extinction learning on c-Fos expression in vmPFC**. To demonstrate that extinction activated neurons in the vmPFC after stress, 28 CUS-treated rats (14 male and 14 female) were assigned to 2 groups (extinction vs tone control). Extinction was conducted 24 h after the last stressor. Rats were sacrificed by perfusion-fixation with 4% paraformaldehyde 2 h after the onset of the 32-min extinction session. Brains were cut into 40-µm coronal sections [33], incubated in a rabbit anti-Fos antibody (1:2000; ABE457, Millipore, Burlington, MA or 226 008, Synaptic Systems, Goettingen, Germany) for 24 h at 4 °C, followed by biotinylated secondary antibody (1:2000; Jackson ImmunoResearch, West Grove, PA), avidin-biotin complex (Vector Laboratories, Newark, CA), and color generated with a nickel-enhanced diaminobenzidine reaction. Slides were scanned and visualized with a 20X objective using a Zeiss AxioObserver inverted microscope (Zeiss Objective Plan-Apochromat) with Zen3.5 (blue edition) software. For each rat, four bilateral coronal sections, from ∼+3.72 mm to +2.76 mm anterior to bregma [33], were reconstructed into 16-bit grayscale files by Huygens Software (Scientific Volume Imaging, Hilversum, Netherlands). Images were manually aligned to a brain atlas [33] to define PL and IL by anatomical landmarks, such as medial oribitofrontal artery, azygous anterior cerebral artery or azygous pericallosal artery. A 1 mm^2^ area of vmPFC, including IL and the ventral portion of PL (Fig. 3a), was analyzed using Fiji ImageJ (NIH). Fos positive cells in the 1 mm^2^ vmPFC area were counted, and the total number of Fos-positive cells were averaged across eight sections to generate a mean number of positive cells per mm^2^ per rat.

**Chemogenetic inactivation of pyramidal neurons in vmPFC**. An inhibitory Gi-coupled DREADD was used to inhibit neuronal activity of pyramidal cells. 115 rats (52 male and 63 female) were assigned to 6 groups defined by stress (CUS vs NS), and extinction + Gi DREADD treatment (tones+GFP, extinction+GFP, extinction+Gi DREADD). Fear conditioning was performed 7-10 days after virus injection. CUS began the day after fear conditioning (Fig. 3c). One day after the end of CUS, rats received an injection of the DREADD ligand CNO (1 mg/kg in 0.5% DMSO, i.p.) followed by extinction learning 30 min later. Rats were then tested for sucrose preference 24 h after extinction.

**Fig. 4:**
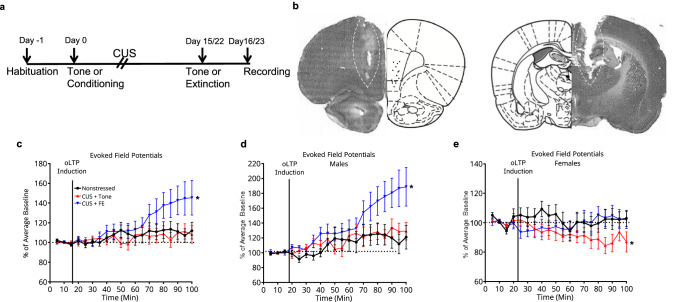
Effects of extinction learning on local field potentials and opto-LTP in the vmPFC. (**a**) Timeline for experiments testing the effects of extinction learning on local field potentials and opto-LTP. (**b**) Representative micrograph showing placement of a recording optrode in the vmPFC (left) and a stimulating electrode in the MDT (right). Brightness and contrast have been optimized to visualize histological detail. (**c**) Fear extinction learning 24 h prior to recording in stressed rats (blue) significantly enhanced the potentiation of MDT-evoked responses by opto-LTP stimulation compared to nonstressed rats (black) or CUS-tone controls (red) (n = 14/group). (**d**) In male rats CUS + fear extinction learning 24 h prior to recording (blue) also significantly enhanced the potentiation of MDT-evoked responses by opto-LTP stimulation compared to nonstressed rats (black) or CUS-tone controls (red) (n = 7/group). (**e**) Female rats that underwent CUS and tone control exposure (red) exhibited a depression of MDT-evoked field potentials after optogenetic stimulation. Nonetheless, optogenetic potentiation of evoked responses was restored to baseline in stressed rats by extinction learning 24 h prior to recording (blue) (n = 7/group). *P < 0.05.

**Effects of extinction on long-term potentiation in the MDT-mPFC pathway after CUS**. 42 rats (21 male and 21 female) were assigned to 3 groups (NS-tone controls, CUS-tones, CUS-extinction). Rats were fear-conditioned or exposed to tones-only, 4 weeks after ChETA viral injection. CUS began the day after fear conditioning. Extinction was conducted 1 day after the end of CUS, and electrophysiological experiments were performed 24 h after extinction (Fig. 4a). Rats were anesthetized using chloral hydrate (400 mg/kg, i.p.) and placed in a stereotaxic apparatus (David Kopf Instruments, Tujunga, CA). Body temperature was maintained at 37 °C. A bipolar stainless-steel stimulating electrode (P1 Technologies, Roanoke, VA) was lowered into the right MDT (AP − 2.5, ML + 0.9, DV –4.6 mm). A tungsten parylene-coated recording electrode (A-M Systems, Sequim WA) was positioned in the ipsilateral vmPFC (AP + 2.5, ML + 0.6, DV –4.0-5.0 mm). An optical fiber affixed to the recording electrode 1 mm above the tip was connected to a 473-nm solid-state laser diode (OptoEngine LLC, Midvale, UT) with 13-15 mW output. Local field potentials were recorded in vmPFC (low cutoff filter 0.3 Hz, high cutoff 1000 Hz) and digitized (Power Lab; AD Instruments, Colorado Springs, CO). A current-response curve was established by stimulating MDT with 30 pulses (100–600 μA in 100 μA steps, 260 µsec pulse width, 0.1 Hz) as described [27]. After a stable baseline was established at 50% maximum response for 15 min, opto-LTP was induced by high frequency laser stimulation of MDT-vmPFC terminals (10 ×1 s trains, 1 ms pulse width, 250 Hz, once every 10 sec for 90 sec), as described [26]. Responses evoked by electrical stimulation of MDT were then recorded for 90 min (6 traces/min), calculated as percent of mean baseline, and analyzed in 5-min bins. Experimenter was blind to treatment.

Immunohistochemistry was performed to confirm ChETA expression in the MDT injection site and in terminals in mPFC. Rats were sacrificed via rapid decapitation. Brains were post-fixed in 4% PFA, and 40 μm sections incubated in primary rabbit anti-GFP antibody (1:5000; Cell Signaling, Danvers) followed by HRP-linked CY3-conjugated secondary antibody (1:1000; Millipore) and counterstained using DAPI. Alternate sections were used to confirm electrode placements histologically. Animals with misplaced electrodes were excluded from analysis.

### Statistical analysis

Datasets were tested for normality and homoscedasticity. Parametric data were then analyzed by 2-tailed t-test or analysis of variance (ANOVA). Overall effects were first analyzed with male and female rats combined, with sex included as a factor. Secondary analyses were then conducted on male and female data separately, as per NIH guidance to analyze and report results after disaggregating by sex, although the experiments were not explicitly powered for these analyses. Fear conditioning, extinction, and locomotor activity were analyzed by 2- or 3-way repeated-measures ANOVA. Stimulus-response curves for evoked potentials were analyzed using an extra sum-of-squares F-test. Post hoc comparisons were made using the Holm–Sidak test. Significance was determined at *p* < 0.05.

## Results

### Antidepressant-like effects of extinction on the forced swim test

The FST detects potential antidepressant efficacy with predictive validity [34, 36]. Prior to testing, there were no differences in extinction between groups with or without swim exposure (*F*_(1, 40)_ = 1.206, *P* = 0.279), between males and females (*F*_(1, 40)_ = 0.499, *P* = 0.488; Fig. 1b), nor any interaction between sex and swim exposure (*F*_(1, 40)_ = 0.057, *P* = 0.812) or between tones, sex and swim exposure (*F*_(15, 600)_ = 0.584, *P* = 0.888). In the 5-min test, immobility was significantly decreased in the extinction group compared with tone controls (*F*_(1, 20)_ = 13.68, *P* < 0.01; Fig. 1c). Immobility was lower overall in females compared to males (*F*_(1, 20)_ = 9.733, *P* < 0.01), consistent with a previous study [37], but extinction decreased immobility in both males (*t*_(10)_ = 2.756, *P* < 0.05) and females (*t*_(10)_ = 2.469, *P* < 0.05; Fig. 1c). To rule out an effect of fear conditioning alone on the FST, immobility in the first 5 min of the pre-test was analyzed, with comparable immobility in tone-control and fear-conditioned rats (*t*_(22)_ = 0.875, *P* = 0.391; Fig. 1d). Similar to antidepressant drugs [34, 38], there was no effect of extinction administered 24 h prior to the pre-swim on immobility in the test swim (tone: 209.63 ± 18.45 sec; extinction: 200.6 ± 20.59 sec; *t*_(10)_  =  0.298, *P* = 0.772). Extinction had no effect on locomotor activity (*F*_(1, 23)_ = 0.150, *P* = 0.7018; Fig. 1e). Distance travelled was comparable (*t*_(25)_ = 0.851, *P* = 0.189; Fig. 1e inset). To determine if the antidepressant-like effects of extinction were lasting, a test session was performed 7 days after extinction (Fig. 1f). Immobility was significantly decreased in the extinction group compared with tone controls (*F*_(1, 23)_ = 18.52, *P* < 0.001; Fig. 1f). Immobility was again lower overall in females (*F*_(1, 23)_ = 9.289, *P* < 0.01). Extinction reduced immobility comparably in males (*t*_(13)_ = 2.437, *P* < 0.05) and females (*t*_(10)_ = 4.635, *P* < 0.001; Fig. 1f). These results indicate that extinction learning has antidepressant-like effects in the FST that are sustained for at least 7 days.

### Antidepressant-like effects of extinction on sucrose preference

The effect of extinction on CUS-induced reduction in sucrose preference was evaluated as a model of anhedonia [39]. Because of the different time required for CUS treatment to achieve similar behavioral effects in male and female rats [16, 26], extinction was performed 15 or 22 days after fear conditioning for male and female rats, respectively (Fig. 2a). Extinction was comparable in NS and CUS groups, in females and males (Stress: *F*_(1, 25)_ = 0.006, *P* = 0.939; Sex: *F*_(1, 25)_ = 1.666, *P* = 0.209; Fig. 2b), with no interaction between sex and stress (*F*_(1, 25)_ = 0.373, *P* = 0.541) or between tones, sex and stress (*F*_(15, 375)_ = 0.901, *P* = 0.563), as shown previously [14, 15, 40]. In nonstressed rats, there was no difference in sucrose preference between tone-control and extinction groups (male: tone 84.0 ± 2.8% vs FE 86.3 ± 5.0%; female: tone 90.5 ± 3.8%; extinction 93.4 ± 0.9%). Therefore, these rats were combined into a single NS control group. There was a significant group effect (*F*_(2, 51)_ = 8.976, *P* < 0.001; Fig. 2c) with no effect of sex (*F*_(1, 51)_ = 1.074, *P* = 0.305). Post hoc tests revealed that CUS decreased sucrose preference compared to NS controls (*P* < 0.01). Extinction reversed the CUS-induced decrease in sucrose preference (*P* < 0.01). As per NIH mandate, data were then analyzed by sex separately. There was a significant effect in males (*F*_(2, 25)_ = 6.862, *P* < 0.01; Fig. 2c). Sucrose preference was decreased by CUS in tone controls (*P* < 0.05), and extinction reversed the CUS-induced decrease (*P* < 0.01). In females, there was a significant group effect (*F*_(2, 26)_ = 3.640, *P* < 0.05; Fig. 2c). Sucrose preference was decreased after CUS in the tone-control group (*P* < 0.05). In stressed females, there was a moderate increase in sucrose preference after extinction, but it was not significant (*P* = 0.346). However, the CUS-extinction group also did not differ from nonstressed controls (*P* = 0.346), indicating a similar but less robust effect of extinction in stressed females than in males, although it must be emphasized that there was no effect of sex in the primary ANOVA, and this experiment was not explicitly powered to analyze the sexes separately.

### vmPFC activity is necessary for antidepressant effects of extinction

To determine if antidepressant-like effects of extinction were associated with activation of vmPFC in stressed rats, we assessed Fos expression after extinction in stressed rats. Two way ANOVA indicated no effect of sex (*F*_(1, 24)_ = 0.032, *P* = 0.860) and no interaction between sex and extinction (*F*_(1, 24)_ = 0.2154, *P* = 0.6468). Extinction increased Fos expression in vmPFC (*t*_(26)_ = 3.619, *P* = 0.0013, Fig. 3b). After disaggregating by sex, similar effects were observed in males (*t*_(12)_ = 2.500, *P* = 0.027, Fig. 3b) and females (*t*_(12)_ = 2.503, *P* = 0.028; Fig. 3b).

To determine the role of vmPFC activation in the antidepressant-like effects of extinction, a Gi-DREADD was used to inhibit pyramidal cell activity in vmPFC during extinction. Figure 3d shows the location of viral administration into vmPFC. Fear conditioning was unaltered by viral injections in vmPFC (*F*_(1, 71)_ = 1.553, *P* = 0.217, Fig. 3e). Extinction was comparable in NS and CUS groups (*F*_(1, 71_ = 2.125, *P* = 0.149, Fig. 3e) and in Gi- and GFP-controls (*F*_(1, 71)_ = 0.349, *P* = 0.556; Fig. 3e), as shown previously [15]. For sucrose preference, measured 24 h after extinction, there were significant main effects of treatment (*F*_(2, 109)_ = 5.336, *P* < 0.01); stress (*F*_(1, 109)_ = 26.370, *P* < 0.001); and a stress x treatment interaction (*F*_(2, 109)_ = 8.842, *P* < 0.001, Fig. 3f). In GFP-tone controls, CUS decreased sucrose preference (*P* < 0.01). Extinction reversed this effect in GFP-expressing rats (*P* < 0.05). Gi-DREADD inhibition of pyramidal neurons in vmPFC during extinction prevented its beneficial effects in reversing the CUS-induced reduction in sucrose preference 24 h after extinction (*P* < 0.001 compared to CUS-GFP-extinction), suggesting the antidepressant-like effects of extinction require activation of pyramidal neurons in vmPFC during extinction. Three-way ANOVA indicated a significant main effect of sex (*F*_(1, 103)_ = 10.77, *P* < 0.01), in that females showed higher sucrose preference overall than males. However, there were no interactions of sex x stress (*F*_(1, 103)_ = 0.015, *P* = 0.903), sex x treatment (*F*_(2, 103)_ = 0.741, *P* = 0.479), nor sex x treatment x stress (*F*_(2, 103)_ = 0.829, *P* = 0.439). In males, there was a significant effect of stress (*F*_(1, 46)_ = 10.94, *P* < 0.01); a treatment x stress interaction (*F*_(2,46)_ = 3.642, *P* < 0.05); and a near-significant main effect of treatment (*F*_(2,46)_ = 3.057, *P* = 0.056; Fig. 3f). Post-hoc tests indicated that sucrose preference was decreased by CUS in tone controls (P < 0.05). Extinction reversed the effect of stress (P < 0.05) and that was prevented by Gi-DREADD inhibition of vmPFC during extinction (P < 0.05). In females, there was a significant effect of stress (*F*_(1, 57)_ = 17.31, *P* < 0.001) and interaction of stress x treatment (*F*_(2,57)_ = 6.821, *P* < 0.01) and a near-significant effect of treatment (*F*_(2,57)_ = 2.998, *P* = 0.058; Fig. 3f). As in males, post-hoc tests indicated that Gi-DREADD inhibition of vmPFC during extinction in females prevented its therapeutic effect on sucrose preference after CUS (P < 0.001).

### Extinction enhances synaptic plasticity in vmPFC

The MDT-mPFC pathway is affected by CUS [26, 27]. To test if behavioral effects of extinction after CUS were accompanied by changes in plasticity in the MDT-mPFC pathway, we measured optogenetic potentiation of MDT-evoked responses in the vmPFC after CUS and extinction learning (Fig. 4a) A stimulating electrode was placed in the MDT and responses were recorded in the vmPFC (Fig. 4b). Extinction enhanced optogenetic potentiation of MDT-evoked responses in stressed rats compared to nonstressed and CUS-tone controls (*F*_(4, 834)_ = 18.27, *P* < 0.001; Fig. 4c). Although extinction enhanced optogenetic potentiation of MDT-evoked responses in both sexes, analyzing separately by sex revealed nuanced differences. In males, extinction facilitated optogenetic potentiation in stressed rats compared to nonstressed and CUS-tone controls (*F*_(4, 414)_ = 19.84; *P* < 0.001; Fig. 4d). In control females, optogenetic stimulation only modestly potentiated evoked responses compared to males, and in stressed females, optogenetic stimulation induced a slight depression rather than potentiation of evoked responses (*F*_(4, 834)_ = 18.27, *P* < 0.0001; Fig. 4e). Nonetheless, extinction still reversed this attenuated response in stressed females.

## Discussion

Fear extinction, administered as a therapeutic intervention in rats, had antidepressant-like effects. Extinction reduced immobility in the FST and reversed the CUS-induced decrease in sucrose preference. Extinction learning activated neurons in the vmPFC of stressed rats, and inactivation of pyramidal neurons in vmPFC during extinction prevented its antidepressant-like effects. Extinction enhanced optogenetically-induced potentiation of MDT-evoked responses in vmPFC of stressed rats, particularly in males. These results indicate that activity-dependent neuroplasticity induced by extinction in vmPFC is involved in its antidepressant-like effects after chronic stress.

During exposure therapy, patients are repeatedly exposed to a fearful memory in a safe environment to reduce negative emotional responses [41]. Exposure therapy has also been effective in treating depressive symptoms [11, 42]. In the current study, extinction decreased immobility in the FST 24 h and 7 days after extinction, suggesting that the antidepressant effects are relatively long-lasting. Similar to antidepressant drugs [34, 38], the effects of extinction depend on having had prior exposure to the FST, as there was no effect if extinction was administered 24 h prior to the first swim. Antidepressant-like effects of extinction were further demonstrated using the sucrose preference test. Reduced sucrose preference after CUS, modeling anhedonia [39], is reversed by chronic treatment with traditional antidepressants [35, 43, 44] and acutely-acting antidepressants [45, 46]. Extinction reversed the CUS-induced reduction of sucrose preference tested 24 h after extinction. Although stress can impair extinction [47], we and others have shown that when conditioning occurs before stress, extinction itself is unaffected [14–16, 40], and it was comparable in the present study in rats with and without CUS. These results suggest that the antidepressant-like effects of extinction are fast-acting and long-lasting, similar to those of acutely-administered antidepressants, such as ketamine [48].

Hypoactivity in the PFC has been reported in patients with MDD [49, 50]. Deep brain stimulation of the vmPFC has antidepressant efficacy in treatment-resistant patients [51]. In rodents, electrical or optogenetic stimulation of mPFC has antidepressant-like effects on the FST and sucrose preference test [52–54]. The role of vmPFC in extinction is well established [55]. Increased mPFC activity is associated with expression of extinction memory [56]. In agreement with past studies [57], we found that extinction activated neurons in vmPFC of stressed rats. Further, inhibition of neurons in vmPFC during extinction prevented its therapeutic effects on CUS-induced anhedonia. Again, extinction itself was not altered by this inhibition, as we and others have reported [14–16, 58]. These results suggest that plasticity underlying antidepressant-like effects of extinction requires activation of pyramidal neurons in vmPFC, as we have also shown previously for the effects of extinction on other behaviors compromised by CUS [14–16]. Similar to extinction, ketamine, which increases glutamate signaling in the PFC in healthy subjects and patients with MDD [59] and elevates activity of pyramidal cells in the vmPFC of rodents [60], produces antidepressant actions lasting from hours up to 1 week [61, 62]. Moreover, inactivation of vmPFC by muscimol, a GABA-A receptor agonist, blocked the antidepressant effects of ketamine 24 h later [54, 63]. These results suggest that plasticity produced by an optimal level of vmPFC activation is associated with antidepressant-like effects of both ketamine and extinction.

Although the bulk of evidence suggests hypoactivity in vmPFC in depression [64], there are reports of hyperactivity [65], which also disrupts processes related to emotional regulation [66]. It is possible that different subpopulations of neurons in mPFC, defined by phenotype or specific projection targets, may be affected differently, and which effect predominates (i.e., hypo- or hyper-activity) may be related to different subtypes of depression [67, 68]. Previous work investigating afferent-evoked responses in mPFC suggests that rather than hypoactivity per se, stress-induced cognitive deficits may be related more to hypo-responsivity of the mPFC to specific afferent inputs [26]. It has also been suggested that chronic stress can lead to hyperactivity specifically of GABAergic interneurons in mPFC [69], disrupting the excitatory/inhibitory balance. This might account for both the increased basal metabolic signal reported in some studies, and due to the subsequent inhibition of glutamatergic projection neurons, the attenuated responsivity of mPFC to specific afferent inputs. Diminished vmPFC activity linked with exaggerated amygdala reactivity was observed in patients with depression and PTSD with high levels of negative affect [70], consistent with the idea that specific sub-populations of mPFC neurons may be affected, contributing to specific symptom domains.

The mPFC receives excitatory afferents from the thalamus, hippocampus and amygdala [71]. The MDT-mPFC pathway is particularly vulnerable to stress [26, 27], and direct activation of this pathway decreased depression-related behaviors [72]. In treatment-resistant depression, connectivity between PFC and thalamus is reduced [73, 74], and antidepressant response to transcranial magnetic stimulation is associated with increased MDT-mPFC connectivity [74, 75]. To investigate whether extinction enhanced plasticity in the MDT-vmPFC pathway of stressed rats, we tested optogenetic potentiation of MDT-evoked field potentials in vmPFC. In unstressed rats, optogenetic potentiation of MDT-evoked responses in vmPFC was greater in males than in females. CUS attenuated optogenetic potentiation of evoked responses in females, even converting it to a depressed response, with little effect in males. However, despite differences at baseline and after stress, extinction enhanced optogenetic potentiation of MDT-evoked responses in both males and females after CUS, indicating facilitation of activity-dependent plasticity. Enhanced potentiation by extinction in stressed male rats compared to both unstressed controls and females may account for the greater beneficial effect of extinction on sucrose preference in males after CUS. However, although 2 weeks CUS in males and 3 weeks in females produce comparable behavioral impairment in several measures, it is also possible that the different duration of stress treatment may be a factor in the differential effects on optogenetic potentiation. This remains to be investigated.

Reduced cortical volume in PFC has been observed in MDD [18], and greater PFC volume predicted better outcomes and lower depressive symptoms in MDD [17]. Stress has been shown to decrease dendritic length of apical dendrites on mPFC pyramidal neurons [22–25]. Acute administration of the rapidly-acting antidepressant, ketamine, has been shown to reverse stress-induced dendritic retraction in the PFC [45]. MDT inputs preferentially activate neurons in layer II/III [76], and stress reduced dendritic length in layer II/III pyramidal cells [77, 78]. Layer V pyramidal neurons in vmPFC also receive MDT input [76], and project to subcortical targets that mediate stress-related behaviors. Optogenetic activation of layer V pyramidal neurons in PFC induced antidepressant-like effects [79]. Thus, reversal of CUS-induced dendritic pruning in pyramidal neurons may contribute to the enhanced optogenetic potentiation of MDT afferent input to vmPFC after extinction.

Exposure therapy is effective in reducing symptoms of both PTSD and depression, improving cognitive flexibility, reducing perseveration and negatively biased thought, and improving adaptive responding [80, 81]. Similar to the effects of exposure therapy in humans [82], in this and in previous studies, we showed that extinction learning as an animal model of exposure therapy rescued stress-induced cognitive deficits, avoidance behavior, and anhedonia, and showed long-lasting antidepressant-like efficacy in the FST [14, 15]. Not all of these therapeutic effects of extinction are a direct result of Pavlovian extinction of the predictive value of the conditioned stimulus in signaling the unconditioned stimulus. Rather, our current and previous studies have shown that plasticity induced by extinction learning in the vmPFC restores optimal functioning [14–16, 83] responsible for the modulation of prefrontal-related symptom dimensions [84–87]. Subpopulations of neurons in vmPFC innervate cortical and subcortical targets involved in a range of behavioral, affective, and cognitive responses associated with depression and PTSD [17, 88]. For example, previous evidence shows that a projection from the mPFC to the dorsal raphe nucleus regulates antidepressive-like responses in the FST [89]. A projection from the vmPFC to nucleus accumbens regulates hedonic behaviors [90]. And we have shown that a projection from vmPFC to the lateral septum modulates active vs avoidant coping behavior [91]. Further experiments are ongoing to determine the specific top-down neural circuit mechanisms responsible for the range of behavioral effects of extinction learning after stress. Also, because exposure therapy can sometimes be context-specific, identifying ways to not only strengthen the plasticity induced by extinction in the vmPFC, but also to strengthen the modulatory influence of the vmPFC on downstream targets mediating non-associative symptoms would be beneficial.

In summary, this study demonstrates that extinction learning, administered as a therapeutic intervention, has antidepressant-like effects on two behavioral measures, immobility on the FST and reduced sucrose preference after CUS. This further validates and generalizes extinction as a rodent model of cognitive-behavioral therapy, specifically exposure therapy, to study neurobiological mechanisms underlying its beneficial effects. Extinction facilitated adaptive plasticity in the vmPFC, similar to the effects of fast-acting antidepressants [48, 92]. This supports reports that exposure therapy, an effective treatment for PTSD, may also be effective for symptoms of depression [9–11], especially for individuals with PTSD comorbid with depression. Identifying mechanisms responsible for the therapeutic effects of extinction may suggest potential strategies to enhance those processes and improve the efficacy of exposure therapy.

## Data Availability

Primary data included in the results reported in this study are available after publication from the corresponding author upon reasonable written request.
